# Identification of an intronic *Alu* insertion in the *SYNE1* gene associated with autosomal recessive spinocerebellar ataxia type 8

**DOI:** 10.1016/j.gimo.2024.101893

**Published:** 2024-09-10

**Authors:** Maryse Gagnon, Nadia Bouhamdani, Dimiter P. Kolev, S. Hussain Askree, Mouna Ben Amor

**Affiliations:** 1 Université de Sherbrooke, Sherbrooke, QC, Canada; 2 Centre de formation médicale du Nouveau-Brunswick, Moncton, NB, Canada; 3 Vitalité Health Network, Moncton, NB, Canada; 4 Université de Moncton, Department of Chemistry and Biochemistry, Moncton, NB, Canada; 5 Atlantic Cancer Research Institute, Moncton, NB, Canada; 6 MNG Laboratories (Medical Neurogenetics, LLC.), a Labcorp subsidiary, Atlanta, GA

**Keywords:** SYNE1, Nesprin, SCAR8, Alu elements, retrotransposons

The spectrin repeat-containing nuclear envelope protein 1 (*SYNE1; HGNC:17089*) gene, which is located on chromosome 6q25 and contains 146 exons, encodes protein isoforms nesprin-1 and nesprin-2, both of which are important for maintaining the structure and function of the cerebellum.^1^^,^^2^ Variants in *SYNE1* are associated with slowly progressive cerebellar syndromes with an adult onset, including autosomal recessive cerebellar ataxia type 1 or spinocerebellar ataxia type 8 (ARCA1/ SCAR8, [OMIM: 610743]), also known as ataxia of Beauce, named after the region in Quebec, Canada, with high prevalence of the disease.^1^^,^^3^ SCAR8 remains the most common manifestation of *SYNE1* deficiency described at present and is characterized by slowly progressive ataxia, dysarthria, eye movement abnormalities, and occasionally, sensory neuropathy, upper and/or lower motor neuron dysfunction, extrapyramidal signs, brainstem findings, and cognitive features.^4^

A 41-year-old female patient was referred to the medical genetics clinic by her neurologist for evaluation of late onset of balance and fine motor difficulties, dysarthria, and cerebellar degeneration on brain imaging. The patient had onset of balance issues and clumsiness after giving birth in 2013. Five years later, at 35 years old, the patient developed progressive dysarthria and fine motor dysfunction, with worsening balance and recurrent falls. There was no report of cerebellar dysfunction or of a diagnosis of ataxia in relatives as per the family history. The patient’s parents were of mixed background, being French Canadian from Quebec and Acadian from New Brunswick. Neurological examination showed normal deep tendon reflexes, normal muscle strength, and no significant dysmetria or dysdiadochokinesia. The patient’s gait, however, was narrow and unstable, unilaterally drifting to the left, with difficulties walking on tip toes and heels. The patient had difficulty in medial convergence bilaterally, but extraocular movements were otherwise within normal range, with no obvious nystagmus.

Brain magnetic resonance imaging in March 2021 revealed isolated cerebellar atrophy, affecting mainly the vermis, and brain single photon emission computed tomography scan combined with a computed tomography scan in June of the same year showed significant abnormality in the cerebellum. Additional testing included a microarray for chromosomal anomalies, blood gas, lactate, and ammonia, which were all normal. Plasma amino acids, urine organic acids, very-long-chain fatty acids, sterol profile, and serum screen for congenital defects of glycosylation did not reveal any significant findings.

A targeted exome to the patient’s phenotype was performed, using Blueprint Genetics (BpG) Whole Exome Plus Test (Supplemental Figure 1). Results revealed that the patient was heterozygous for 2 variants in the *SYNE1* gene: NM_033071.3:c.1371+2del (Chr6: 152,804,217; GRCh37), classified as a likely pathogenic variant, and NM_033071.3:c. 9168-33_9168-32insAlu (Chr6: 152,697,725; GRCh37), classified as a variant of uncertain significance because retrotransposable insertion in the *SYNE1* gene had neither been previously described in the medical literature nor been reported in disease-related variation databases. The Alu insertion is of unknown length; however, our bioinformatics team was able to estimate the size of the Alu insertion to be between 200 and 300 bp (NG_012855.2 (NM_033071.3): c.9168-33_9168-32insN[250]).

The *SYNE1* NM_033071.3:c. 1371+2del variant was inherited from the patient’s father, who has Quebec and Acadian ancestry, whereas the *SYNE1* NM_033071.3:c. 9168-33_9168-32insAlu variant was inherited from the mother, an Acadian from New Brunswick.

An RNA sequencing targeted analysis, specific for the *SYNE1* gene, was completed at MNG Laboratories using next-generation sequencing technology on the patient’s blood sample. This testing revealed aberrant splicing around the location of both identified variants (Figure 1A and B). In the intron 57 region where the NM_033071.3:c.9168-33_9168-32insAlu variant was detected, an aberrant splice junction was recognized in approximately 50% of the sequenced transcript, extending from the splice donor site of exon 57 to the splice acceptor site of exon 59, congruous with the skipping of exon 58 on one of the alleles (Figure 1A). An estimated half of total transcripts in this region presented a canonical splice junction across intron 57 and intron 58. These anomalies are predicted to cause a frameshift variant and a premature termination of the nesprin-1 and nesprin-2 proteins. For the NM_033071.3:c.1371+2del variant located at the exon 14/intron 14 boundary, approximately 25% of the transcripts demonstrated the inclusion of 57 bases of intron 14 and used GT at location chr6:152,804,161-152,804,162; GRCh37 as a novel aberrant splice donor site. These reads extended from this aberrant splice junction within intron 14 to the canonical splice acceptor site of exon 15 (Figure 1B). These aberrant transcripts are predicted to result in a frameshift variant with a premature termination of the resultant nesprin-1 and nesprin-2 proteins. In addition, canonical splice junction for intron 14 (exons 14-15) was also observed in roughly 75% of the total transcripts located in this region. The *SYNE1* variant NM_033071.3:c. 9168-33_9168-32insAlu was consequently reclassified as likely pathogenic by the Blueprint Laboratory team.

**Figure 1:**
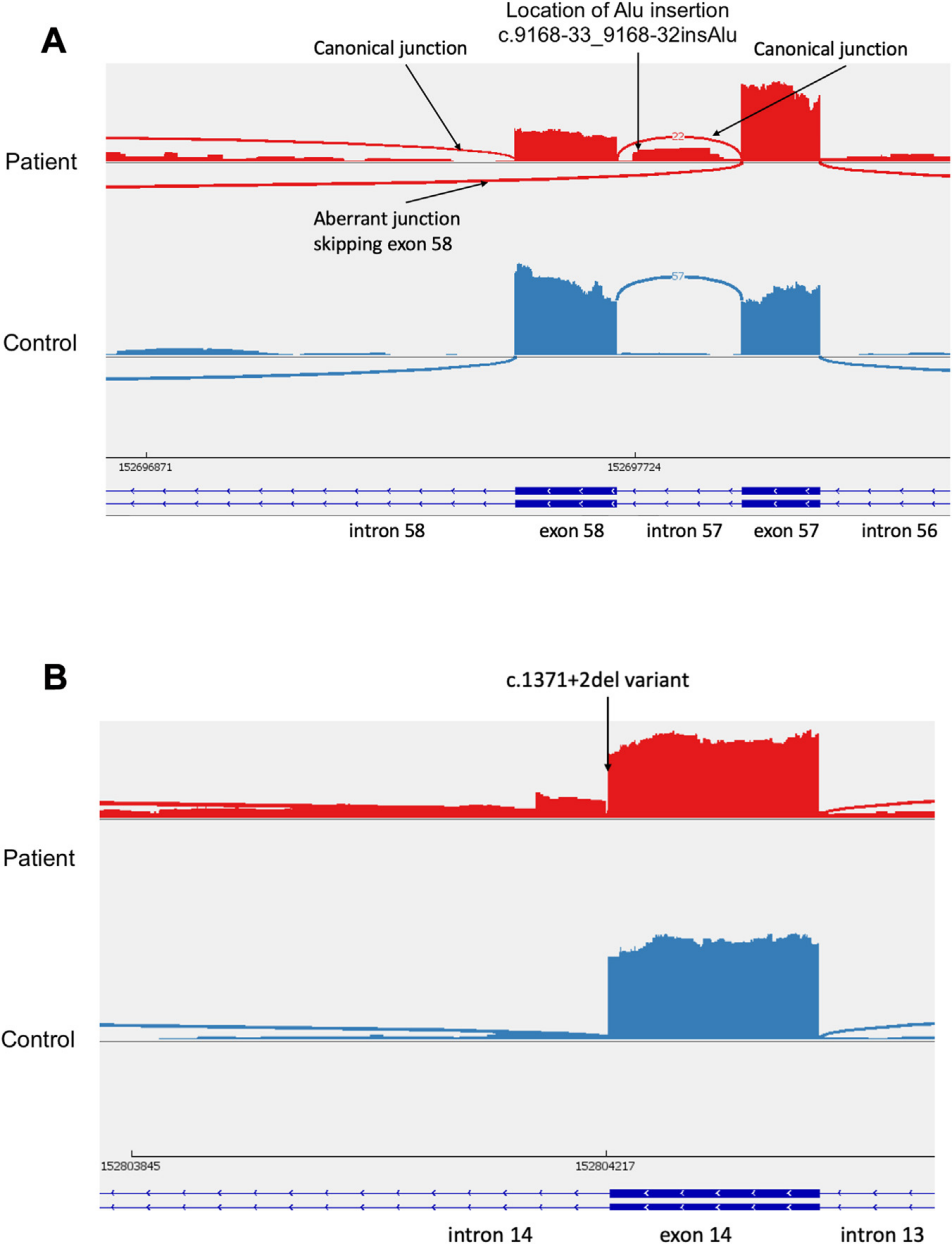
**Results of RNA sequencing in *SYNE1* demonstrating aberrant splicing in both variants of interest**. A. Aberrant splice junction in intron 57 region, where the NM_033071.3:c.9168-33_9168-32insAlu variant is located. B. Aberrant splice junction at the exon 14/intron 14 boundary, where the NM_033071.3:c.1371+2del variant is located.

To our knowledge, this case is the first to report a disease-causing intronic insertion of a retrotransposable element, or *Alu* repeat, in the *SYNE1* gene. Based on the clinical phenotype, the biparental inheritance of both *SYNE1* variants, and the altered splicing as revealed by the RNA studies, the patient received the diagnosis of spinocerebellar ataxia type 8 (SCAR8, OMIM: 610743). Recommendations regarding the management and symptomatic treatment options of *SYNE1*-deficient cerebellar ataxia were provided to the patient’s neurologist and family doctor. Genetic counseling and carrier screening for the *SYNE1* variants was also made available to at-risk family members.

Retrotransposable insertions are discrete sequences of DNA that are part of a group of genes that can move from a site to another within a genome and sometimes between different genomes.^4^ Retrotransposons, which can contain long terminal repeats, duplicate using reverse-transcribed RNA intermediates and are then inserted at new locations in the genome.^4^ Non-long terminal repeat retrotransposons including long interspersed nuclear element 1, *Alu*, and short interspersed nuclear elements-variable number of tandem repeats-Alu elements, are the only transposable elements proven to be currently active in humans.^4^ Active transposable elements can cause various diseases by merging into genes.^5^ In fact, approximately 130 genetic disorders have been linked to de novo retrotransposable insertions,^6^ including hemophilia A, neurofibromatosis type 1, cystic fibrosis, Duchenne muscular dystrophy, β-thalassemia, and cancer.^7^, ^8^, ^9^ SCAR8 can now also be added to this list.

This new finding may help enhance our understanding of disease mechanisms and foster future therapeutic avenues targeting different cerebellar ataxias. Being somewhat highly prevalent in nearby Quebec, the frequency of SCAR8 in Acadian population in New Brunswick might be underestimated. Therefore, targeted populational screening of *SYNE1* variants might be warranted in individuals of French-Canadian ancestry presenting with cerebellar dysfunction.

## Conflict of Interest

The authors declare no conflicts of interest.

## Figures and Tables

**Supplementary Figure 1:**
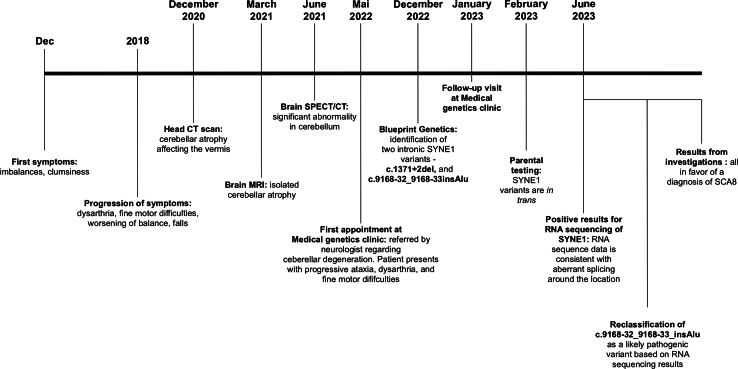
Timeline of events leading up to the diagnosis of SCAR8 in this patient.
